# Interactions of protective behavioral strategies and cannabis use motives: An online survey among past-month users

**DOI:** 10.1371/journal.pone.0247387

**Published:** 2021-03-01

**Authors:** Gregor Genrich, Céline Zeller, Hans Jörg Znoj

**Affiliations:** Department of Health Psychology and Behavioral Medicine, University of Bern, Bern, Switzerland; Brown University, UNITED STATES

## Abstract

Given the constant high prevalence of cannabis use and cannabis dependence, it is important to determine protective behaviors on the individual level, which buffer the effects of risk factors. Protective Behavioral Strategies for Marijuana (PBSM) have been identified to play an important role for harm reduction in adolescent and young adult users. In the present study, we analyzed if PBSM moderate the effects of use motives (captured by the Marijuana Motives Measure, MMM) on the severity of dependence beyond the effects of age, gender, education and cannabis use frequency. We used confirmatory factor analysis (CFA) to validate the German versions of PBSM and MMM. Data was gathered in an online survey distributed to randomly chosen households in the city of Bern in the German speaking part of Switzerland. The final sample comprised 362 past-month users. Results showed negative correlations between PBSM and cannabis use frequency and severity of dependence. The only motives being correlated with severity of dependence were coping and routine, beyond frequency of use. PBSM significantly moderated the effect only of routine motives on the severity of dependence. However, only a few cases who used PBSM extensively were affected. PBSM appear to be an important factor to reduce harm among past-month users but not among those with dependent use patterns, e.g. coping and routine users. Clinical implications are discussed. The routine factor adds significantly to the MMM and should be implemented and improved in future studies. PBSM as well as the MMM can be used in future studies in German speaking populations.

## 1. Introduction

Cannabis is the most often used illegal psychoactive substance worldwide [1]. In Switzerland, 7.3% of the population used cannabis at least once in the past year, which is near the European average. 3.1% reported past-month use (current users), that is more than threefold in adolescents and young adults, and nearly threefold in men compared to women [2]. Most current users used cannabis one to two times per week and more than 20% were heavy users with daily or near daily use; this distribution is comparable to that of the US [3]. On the one hand, if asked about the effects of cannabis use, recreational users frequently report enhanced sociability and sensitivity, euphoria, feeling relaxed or experiencing a pleasurable rush or “buzz” [4, 5]. On the other hand, there is a strong frequency- and dose-related connection between cannabis use and negative consequences. Also heavy use (daily or near daily) has consistently shown to be associated with adverse health- and psychosocial outcomes [6]. Several large reviews distillated the current evidence about the effects of cannabis use [1, 6–8]. There is substantial evidence that regular cannabis users have higher risk for myocardial infarction and testicular cancer; mental disorders like psychosis, mania, anxiety and suicide; changes in brain structure and function; cognitive impairment and using tobacco, alcohol and other illegal drugs. However, it is conclusive that a dependence syndrome is the most common known adverse effect of frequent cannabis use. It is estimated that one in ten lifetime users, one in six who initiated use in adolescence, and nearly one in two daily users develop dependence according to DSM-IV criteria for cannabis use disorder (CUD) [9, 10]. Caulkins [11] argues that today’s prevalence of dependence is even higher because of the growing number of heavy users, legalization, reduced risk-perception and higher amounts of tetrahydrocannabinol (THC) in cannabis products in illegal as well as legal markets. Alike, in Switzerland 1.1% of the general population or 94’000 cannabis users report problematic cannabis use, defined as eight points or more on the cannabis use disorder identification test (CUDIT, [12]). That corresponds to 35% of all current users who report past month use [13]. Cannabis has rather low addictive potential compared to alcohol, tobacco and other drugs [14, 15] and reports of treatment seeking patients seem to be less severe than those addicted to alcohol or opioids [16]. Nevertheless, dependent use-patterns are strongly associated with psychosis, poor mental health, cognitive impairment and school failure [17, 18]. Dependent users have psychosocial, occupational and legal problems following the inability to control their cannabis use. They can experience a withdrawal syndrome accompanied by anxiety, insomnia, appetite disturbance and depression [19]. Especially in younger cannabis users, heavy cannabis use has serious effects on brain- and psychosocial development. This also affects public health: continuous high prevalence of cannabis use despite restrictive drug policy and increasing heavy use among adults [20]; globally increasing numbers of treatment and counseling seeking users [1, 21]; the increase of THC concentration in street-cannabis and new highly potent cannabis products following legalization; lower risk-perception and high availability following legal changes in medical and recreational cannabis [22]. These factors are likely to increase the public health costs associated with cannabis dependence.

However, most users are in control of their cannabis use and do not experience any or very limited negative consequences. Additionally, years of repressive regulatory means did not reduce the prevalence of cannabis use or dependence. The fact that there are no signs of a future decrease poses the need for other ways to promote health and reduce harm for regular cannabis users [23]. We further need to assume that most individuals who engage in any kind of risky activities do not want to harm themselves. They want to and do find ways to increase the benefits and reduce or avoid potential harm resulting from such behavior. This is also the case with cannabis use, which is not problematic for most users. Instead of focusing on abstinence, it may be more informative to study the actual protective behavior of the majority who is in control of their cannabis use and to look at individual risk factors most prevalent in those with dependent use patterns.

In 2016, the Protective Behavioral Strategies for Marijuana (PBSM) scale was introduced [24]. Stemming from a harm reduction perspective this collection of strategies can be used to asses behaviors employed by regular cannabis users to control their use and limit heavy use as well as negative consequences. It was validated in young adult college students, resulting in acceptable psychometric properties of a one-factor structure for the 36-item long-version and the 17-item short-version [24, 25]. PBSM are robustly associated with lower frequency of cannabis use, less negative consequences [24–29] and less symptoms of dependence [24, 30] in US college students. In veterans and college students with posttraumatic stress disorder (PTSD) who are at risk for experiencing cannabis related negative consequences, the use of PBSM is associated with experiencing less cannabis related problems and less CUD symptoms [28, 30]. Known risk-factors for cannabis use and for experiencing negative consequences, like male sex, facets of impulsivity and cannabis use motives, are less predictive of use frequency in college students who use PBSM above average [26, 31]. However, it remains unclear, how PBSM interact with cannabis use motives in predicting severity of dependence.

PBSM help to understand the characteristics of different ways of cannabis use. Furthermore, they are being discussed as a potential useful point of intervention for behavioral change, shifting the focus from abstinence to controlled use in prevention as well as counselling or therapy interventions [24, 26, 28].

The evidence on individual risk factors that lead to the progression from cannabis use to dependence is rather ambiguous and the direction of their association is always under discussion. It can be seen best as the result of several psychosocial factors in interaction with biographical processes and events. They are not the same as those for cannabis use or abuse. Given the large numbers of non-dependent users, cannabis use frequency does not suffice in predicting cannabis dependence, although it is repeatedly shown to be a major source of variance. Other indicators are being assessed that can explain variance in cannabis dependence beyond frequency of use. As stated in a recent systematic review, early onset of cannabis use (starting at 11–15 years), positive psychotropic effects of cannabis, solitary use independent of social context and prior involvement in other drugs have the strongest evidence to be risk factors for cannabis dependence beyond the effects of use frequency [32]. Mental disorders and critical life events like physical abuse or parental separation further seem to predict the progression to dependence.

Furthermore, the distinct roles of cannabis use motives are being investigated and a growing literature has established their association with several cannabis outcomes mostly in college student populations. 1998, Simons et al. [33] developed the Marijuana Motives Measure (MMM), containing five cannabis use motives: coping (e.g. “to forget my worries”), enhancement (e.g. “because it’s exciting”), social (e.g. “because it helps me enjoy a party”), conformity (e.g. “to fit in with the group I like”), expansion (e.g. “because it helps me to be more creative and original”). Already in the alcohol-addiction literature was shown that coping motives predict use-related problems over and above measures of mere use [34, 35]. Also for CUD and cannabis related problems, several studies found unique contribution of coping motives above the effects of frequency of use and other known predictors [36–40]. Results about the specific contributions of enhancement, social and expansion motives to cannabis dependence are mixed, what could be due to the inclusion of different covariates in primary studies [41]. Drawing on evidence from other lines of research [5, 42, 43], Benschop et al. [36] tested routine motives as a possible sixth factor for the MMM, resulting in an additional 2-Item factor (“out of boredom” and “out of habit”). They argue that motives unique to cannabis may have been overlooked by adapting the measure from the alcohol motive questionnaire. After removing three items from the original MMM because of poor factor loadings, they found the updated six-factor version of the MMM to be internally consistent (Cronbachs’s alpha .68−.85). Routine motives significantly added 4.8% to the explained variance in CUD symptoms (total 13.1%). Only coping and routine motives showed significant associations with CUD [36]. To the best of our knowledge this version of the MMM has not been replicated until now and no version of the MMM is available in German.

### 1.1. Present study

The present study aims to examine the interaction of PBSM and MMM in predicting the severity of dependence in a German-speaking sample that is heterogeneous regarding gender, age and educational level. Pearson [44] and Bresin and Mekawi [41] highlighted the importance to assess indicators that can explain variance in negative consequences of cannabis use above the medium effects of cannabis use frequency. Therefore, it is of special interest in this study to control for cannabis use frequency. It is hypothesized that, while controlling for gender, educational level and cannabis use frequency, PBSM moderate the effect of MMM on the severity of dependence in distinct ways: (1) PBSM-use is significantly negatively associated with the severity of dependence. (2) Motives are associated with severity of dependence in a way that coping motives and routine motives are significant positive predictors; no specific assumption is made about the effects of enhancement, social, conformity and expansion motives. (3) The interaction of PBSM-use and coping motives as well as PBSM-use and routine motives is negatively associated with the severity of dependence.

To the best of our knowledge, PBSM have not yet been translated to German and was only tested on college students and young adult veterans. Therefore, a first translated version of the scale and an evaluation of its psychometric properties in the current sample will be provided. Additionally, a translation of the MMM and an evaluation of its updated factor structure will be provided.

## 2. Method

### 2.1. Participants and procedure

The participants in the current study represent a subset of a larger random sample of the city of Bern in the German speaking part of Switzerland. Data was collected in an online survey that measured psychological constructs as well as opinions about cannabis use and the legal situation of cannabis in users and non-users. The city administration provided a random sample of 6000 household addresses. They were deleted from the university server immediately after sending the letters containing the web address to the survey. In the letter as well as in the online survey, participants were informed that their responses will be collected anonymously and that it will not be possible to trace them back. If participants did not have internet access, they could be provided with a paper-pencil version upon request. Additionally, information and access to the survey were distributed by local online newspaper and the website of the University of Bern. We programmed the questions, supplied the survey and collected the data (April and May 2019) using the online application Qualtrics®. According to the Swiss “Humanforschungsgesetz”, the study was approved by the ethics committee (Kantonale Ethikkommission) of the University of Bern stating that it is in accord with Swiss regulations on research with humans but is not within the regulations of the law (KEK-No Req-2019-00253). By starting the survey, participants consented that their data will be collected anonymously and used only for the purpose of this research project.

A total number of 1303 individuals participated in the survey. Due to small group sizes, participants who were gender-diverse or had no educational degree were excluded from further analysis. The current study focuses on participants who used cannabis at least once per month, disclosed their age, gender, education and cannabis use frequency, so the analytic sample was reduced to 367 participants. After removing five cases with nonsensical data, the final sample consisted of 362 participants. Of those 30.7% were female and the mean age was 32.7 (15 to 79). Educational level was 12.7% High School degree, 30.9% finished apprenticeship, 24% Higher College degree and 32.3% University degree. 21.3% reported occasional, 34.0% moderate and 44.8% heavy use of cannabis. See Table 1 for an overview.

**Table 1:** Demographics of the final sample by contact method.

	Letter		Media		Other		Total	
	n (131)	%	n (148)	%	n (83)	%	n (362)	%
*Gender*								
Male	84	64.1	105	70.9	62	74.7	251	69.3
Female	47	35.9	43	29.1	21	25.3	111	30.7
*Age*								
15–19	9	6.9	9	6.1	3	3.6	21	5.8
20–29	43	32.8	69	46.6	41	49.4	153	42.3
30–39	45	34.3	29	19.6	25	30.1	99	27.3
40–49	16	12.3	17	11.5	7	8.5	40	11.1
50–59	13	9.9	21	14.2	5	6.0	39	10.7
60–69	5	3.8	3	2	1	1.2	9	2.5
70+	-	-	-	-	1	1.2	1	0.3
*Education*^*a*^								
High-school	15	11.5	26	17.6	5	6.0	46	12.7
Apprenticeship	33	25.1	47	31.8	32	38.6	112	30.9
Higher college	40	30.6	32	21.6	15	18.1	87	24.0
University	43	32.8	43	29.1	31	37.3	117	32.3
*Cannabis use*								
Occasional use	33	25.2	29	19.6	15	18.1	77	21.3
Moderate use	43	32.8	57	38.5	23	27.7	123	34.0
Heavy use	55	42.0	62	41.9	45	54.2	162	44.8
*PBSM-use*								
Low	15	11.5	28	19	20	24.1	63	17.5
Average	93	71.0	97	65.5	52	62.7	242	66.9
High	23	17.6	23	15.5	11	13.3	57	15.5

*Note*. PBSM = Protective Behavioral Strategies for Marijuana: low = -1 *SD*, high = +1 *SD*. There are small differences in absolute as well as relative numbers between different methods of contact.

^a^ = The chi-square test for independence was significant only for the relation between contact method and education, χ2 (6, *N* = 362) = 13.86, *p* = .031.

### 2.2. Measures

#### 2.2.1. Protective Behavioral Strategies for Marijuana (PBSM)

Cannabis users employ PBSM “before, during, after, or instead of using marijuana to limit heavy use and minimize potential negative consequences” [23, p. 442]. We measured the strategies using the 36-Item scale [25]. Participants were asked, “Please indicate the degree to which you engage in the following behaviors when using marijuana/cannabis”. (1 = never, 2 = rarely, 3 = occasionally, 4 = sometimes, 5 = usually, 6 = always). Since the scale had never been used in a German-speaking sample, our project group translated and consensually discussed each item. Then they were back translated by a native speaker, not involved in the prior translation. If there were discrepancies between the back translated and the original items, the German items were discussed again and changed if necessary.

Since the PBSM scale had to be translated, and never been used in an age- and education-diverse sample, we used confirmatory factor analysis (CFA) to see if the single-factor structure found by Pedersen et al. [25] would be confirmed in our sample. As fit indices we used the standardized root mean square residual (SRMR), root mean square error of approximation (RMSEA), Tucker-Lewis Index (TLI) and comparative fit index (CFI). The CFA produced a significant χ2 of 2000.74 (df = 594), SRMR = .077 and RMSEA of .081 (90% CI .077 to .085), indicating a substandard fit of the model. After examining modification indices, four covariates between error-terms were added to the model. After modification, the fit indices were acceptable: χ2 = 1683.02 (df = 590), SRMR = .0725 and RMSEA = .072 (90% CI .068 to .076). As expected, due to the large sample size, the χ2 value was large and significant. Instead, SRMR and RMSEA below .08 indicated an acceptable fit [45]. Since the RMSEA of the independence model (.137) was below the threshold of .158, the incremental fit indices TLI and CFI—by definition—were very small, had no valid information and are thereby not reported [46]. An alpha coefficient of .92 was calculated, indicating high internal consistency across all 36 items. As a measure of validity the moderate correlations between the PBSM score and cannabis use frequency (*r* = -0.42) as well as severity of dependence (*r* = -0.39) suggest, that the more often PBSM were used, the less frequent cannabis was used and the less symptoms of dependence were present (see Table 2).

**Table 2:** Bivariate correlations.

	1	2	3	4	5	6	7	8	9	10	11	*M*	*SD*
1. PBSM	*0*.*91*											4.09	0.84
2. Coping	**-0.29**	*0*.*81*										1.70	0.79
3. Enhancement	-0.11	0.10	*0*.*78*									4.16	0.82
4. Social	-0.10	**0.15**	**0.37**	*0*.*84*								2.17	0.89
5. Conformity	0.03	0.03	-0.04	**0.17**	*0*.*74*							1.06	0.23
6. Expansion	-0.11	**0.24**	**0.31**	**0.41**	0.05	*0*.*89*						2.41	1.07
7. Routine	**-0.37**	**0.36**	**0.20**	0.08	0.02	**0.16**	*0*.*64*					2.16	1.00
8. SDS	**-0.39**	**0.43**	0.04	-0.03	-0.03	0.09	**0.56**	*0*.*78*				2.31	2.44
9. Frequency	**-0.42**	**0.36**	0.00	-0.03	-0.10	0.10	**0.49**	**0.49**	*-*			1.23	0.78
10. Gender	0.10	0.12	**-0.20**	**-0.25**	-0.05	**-0.20**	0.02	0.03	0.01	*-*		0.31	0.46
11. Education	0.08	**-0.14**	-0.06	-0.12	-0.11	-0.07	-0.01	-0.05	-0.04	-0.05	*-*	1.76	1.04

*Note*. Bold = *p* < 0.01; PBSM = Protective Behavioral Strategies for Marijuana; SDS = Severity of Dependence Scale; Use frequency: 0 = occasional use, 1 = moderate use, 2 = heavy use; Gender: 0 = male, 1 = female; Education: 0 = High School degree, 1 = Apprenticeship, 2 = higher College degree, 3 = University degree; Cronbach’s alpha are shown in the diagonal.

#### 2.2.2. Marijuana Motives Measure (MMM)

Cannabis use motives were assessed using the Marijuana Motives Measure (MMM). The original five-factor version proposed by Simons, Correia, Carey and Borsari [33] has been evaluated and shown satisfactory levels of internal consistency in young adult cannabis users [47]. Benschop et al. [36] re-evaluated the scale through confirmatory and exploratory factor analysis on a Dutch sample of young adult cannabis users and added routine as a sixth factor with two items (boredom & habit). They found acceptable to good internal consistency (Cronbach’s alpha .68 − .85) and significant low to moderate inter-correlations between routine and the original factors (coping [4], enhancement [4], social [6], conformity [3], expansion [5]). All 24 Items scored on a five-point scale with 1 = almost never/never, 2 = some of the time, 3 = half of the time, 4 = most of the time or 5 = almost always/always. Items were translated using the same procedure as for the PBSM scale.

CFA was used to confirm the six-factor structure of the MMM proposed by Benschop et al. [36]. All variables loaded between .55 and .9 on their respective factor. Fit indices indicated an overall acceptable model fit: χ2 = 531.75 (df = 237), CFI = .919, TLI = .905, SRMR = .061, RMSEA = .059 (90% CI .052 to .065). Internal consistency was acceptable to very good (Cronbach’s alpha between .64 and .89, see Table 2). Coping motives and routine motives were moderately to strongly correlated with cannabis use frequency (*r* = 0.36 & 0.49) and the severity of dependence (*r* = 0.43 & 0.56). The more often coping or routine motives were present, the higher were cannabis use frequency and severity of dependence. Coping and routine motives also showed moderate negative correlations with PBSM (*r* = -0.29 & -0.37), suggesting that PBSM were less often used if coping or routine motives were higher (see Table 2).

#### 2.2.3. Severity of Dependence Scale (SDS)

To measure symptoms of dependence we used the Severity of Dependence Scale (SDS) [48]. It consists of five questions aiming at the psychological aspects of dependence, e.g. impaired control or anxiety/worry about the amount of cannabis used. Steiner, Baumeister and Kraus [49] validated the SDS in a German sample (age 18–64; α = .8) and established a cutoff point of two on a scale from 0 to 15 for cannabis dependence according to DSM-IV criteria. The items score on a four-point scale: 0 = almost never/never, 1 = sometimes, 2 = often, 3 = always/nearly always (item 5: 0 = not difficult, 1 = quite difficult, 2 = very difficult, 3 = impossible). Note that in the present study severity of dependence was not treated as a dichotomous (dependence vs. no dependence) but rather as a continuous dependent variable. This accounts for the fact shown by van der Pol et al. [50] that, due to its psychometric properties, the SDS cannot reliably discriminate between dependent and non-dependent young adult users.

#### 2.2.4. Cannabis use frequency

To assess cannabis use frequency we asked participants if they ever used cannabis in their lives. If the answer was yes, we asked if they ever used cannabis in the past 12 months. If the answer was yes, we asked them how often they had used cannabis in the past 12 months. Response options were ‘less than six times’, ‘once a month’, ‘once a week’, ‘several times per week’, ‘every day’. Participants who selected ‘several times per week’ were asked to indicate on how many days per week they use cannabis on average. Response options were 1 to 7.

### 2.3. Statistical analysis

Results from the missing data analysis indicated that 0.15% (38 out of 26’000) of all data points were missing. They were distributed on 19 cases. There was no variable missing more than 1.7% of its data and no missing data pattern was evident after visual inspection of the SPSS output. Also Little’s chi-square test for the assumption of missing completely at random (MCAR) [51] was not significant, χ2 (1037, *N* = 362) = 1093, *p* = .11. Retaining the null hypothesis in Little’s test indicates that missing values were independent of observed as well as unobserved variables in the dataset. Therefore, it was assumed that subsequent analysis yield unbiased parameter estimates and missing values were replaced by a pooled multiple imputed dataset using SPSS.

To prepare the data for further analysis, one PBSM average sum score, six scores for each of the cannabis use motives and one sum score for the SDS ranging from 0 to 15 were calculated for each participant. Item responses for cannabis use frequency were aggregated into three groups of occasional (once per month), moderate (1 to 4 times per week) and heavy (5 to 7 times per week) users. To test the above-mentioned assumptions about interaction of PBSM and each motive we conducted six independent moderation analysis using the PROCESS macro for SPSS [52], controlling for the respective other motives, gender, educational level and cannabis use frequency. The scores of PBSM, MMM and SDS were standardized via z-transformation prior to analysis. Other covariates were not. Gender was coded 0 = male and 1 = female. Educational level and cannabis use frequency were indicator coded and entered with g– 1 groups in the models, with occasional use and university degree as reference groups for cannabis use frequency and educational level respectively. All analyses were conducted within the SPSS version 25 framework.

## 3. Results

Young adults were clearly overrepresented. This is in line with representative epidemiological data, showing that young adults form the largest group of cannabis users in Switzerland [2]. Also male participants were overrepresented as well as moderate and heavy users. The latter may be due to the greater interest in the study in these user groups. In addition to random sampling, we recruited participants via online media, newspaper and the website of the University of Bern. Therefore, we wanted to make sure that those groups did not differ in terms of demographics, cannabis use and use of PBSM. For all relevant variables a chi-square test of independence was calculated, resulting in one significant difference of educational level between contact methods, χ2 (6, *N* = 362) = 13.86, *p* = .031. As can be seen in Table 1 there was no meaningful pattern of differences evident. Although not significant, it is worth mentioning that there is a trend for young adults responding relatively less to the contact letter compared to online/social media and other sources. See Table 1 for an overview of the sample description.

Table 2 presents a matrix of all relevant bivariate correlations, means, standard deviations and Cronbach’s alpha values. The PBSM mean was 4.09 (*SD* = 0.84) indicating that the majority of the sample used protective strategies between occasionally and usually. The mean for the SDS was 2.31 (*SD* = 2.44), that is above the cutoff of 2 for dependence proposed by Steiner et al. [49]. As expected, cannabis use frequency and SDS showed a moderate to strong correlation of *r* = .49. Also consistent with our assumptions were the moderate negative associations between PBSM and SDS (*r* = -.39) and between PBSM and cannabis use frequency (*r* = -.42) as well as the moderate to strong correlations between coping motives and SDS (*r* = .43) and routine motives and SDS (*r* = .56). The negative correlations between coping motives and PBSM (*r* = -.29) and routine motives and PBSM (*r* = -.37) are indicative of the possibility of an interaction effect between motives and PBSM in predicting severity of dependence.

Relevant results of the moderation analysis are depicted in Table 3. All other results with non-significant interactions can be found in S1 Table.

**Table 3:** Interaction model with routine motives as predictor and PBSM as moderator on severity of dependence.

	β	SE	95% CI	
**Constant**	**-0.333**	**0.117**	**-0.56**	**-0.10**
**Routine motives**	**0.345**	**0.051**	**0.24**	**0.45**
**PBSM**	**-0.130**	**0.047**	**-0.22**	**-0.04**
Routine*PBSM	**-0.099**	**0.042**	**-0.18**	**-0.02**
**Coping motives**	**0.205**	**0.047**	**0.11**	**0.30**
Enhancement motives	-0.047	0.046	-0.14	0.04
Social motives	-0.081	0.048	-0.17	0.01
Conformity motives	-0.019	0.042	-0.10	0.06
Expansion motives	-0.019	0.046	-0.11	0.07
Gender	-0.058	0.094	-0.24	0.13
High School degree^d^	0.090	0.137	-0.18	0.36
Apprenticeship^d^	-0.002	0.104	-0.21	0.20
Higher College degree^d^	-0.034	0.111	-0.25	0.18
**Moderate use**^**d**^	**0.256**	**0.117**	**0.03**	**0.49**
**Heavy use**^**d**^	**0.499**	**0.132**	**0.24**	**0.76**

*Note*. Bold = *p* < .05; β = z-standardized regression weights; PBSM = Protective Behavioral Strategies for Marijuana; Gender was coded male = 0 and female = 1.

^d^ = dummy-variable (reference groups *University degree* and *Occasional use* were not entered in the model).

**Fig 1:**
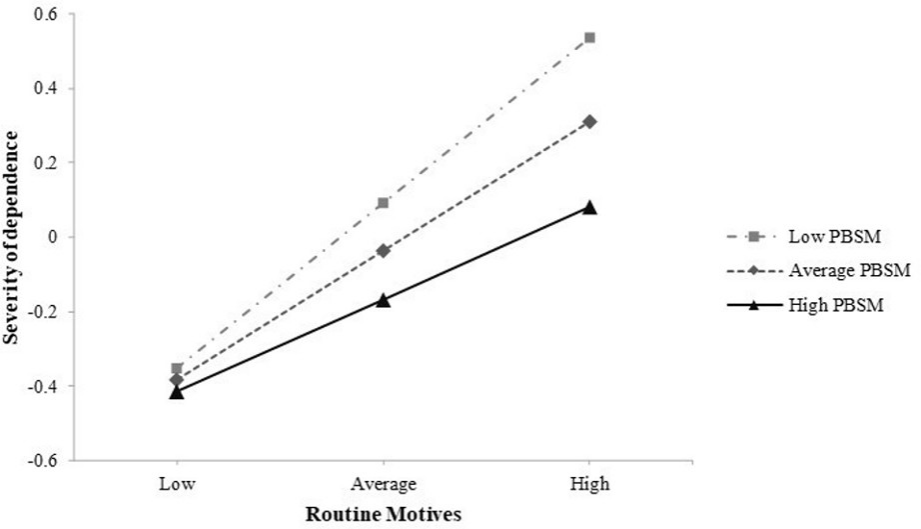
Interaction of routine motives and protective behavioral strategies in predicting severity of dependence. Vertical: z-values for the severity of dependence scale (SDS). Horizontal: z-values for routine motives. The diagonal lines show the regression between SDS and routine motives for different z-values of Protective Behavioral Strategies for Marijuana (PBSM). The association between SDS and routine motives is stronger for lower values and weaker for higher values of PBSM-use.

**Fig 2:**
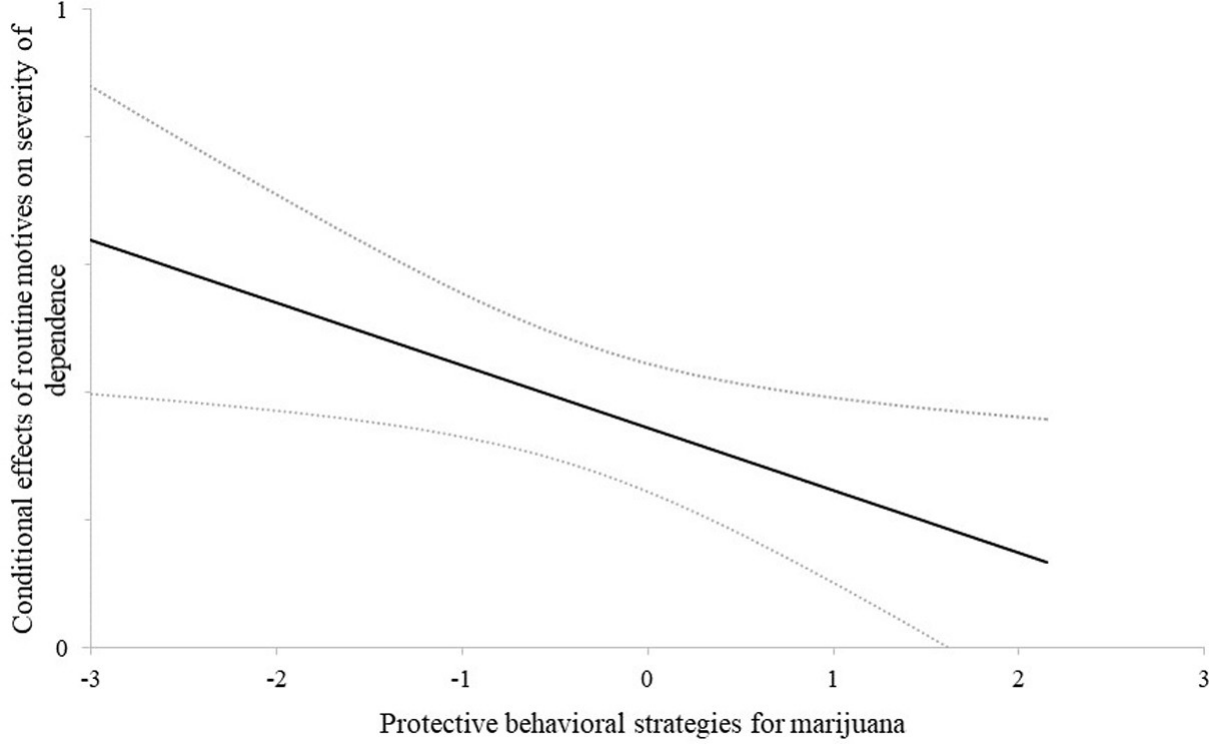
Conditional effects and confidence bands. Horizontal: z-values for protective behavioral strategies for marijuana (PBSM). Vertical: Effect of routine motives on the severity of dependence scale (SDS). The two grey dotted lines are confidence bands and represent the confidence interval for each point on the regression line. If they are not both either above or below zero, the conditional effect of the predictor is no longer significant. The black regression line represents how the use of PBSM affects the association between routine motives and SDS: It is weaker for higher values of PBSM-use. For value below 1.62, routine motives significantly predict SDS. For values above 1.62, this association is no longer significant.

The observed main effects met our assumptions in all six moderation models. Coping motives (β = 0.18; CI 0.1 to 0.3) and routine motives (β = 0.35; CI 0.24 to 0.45) were associated each with higher severity of dependence, independent of other motives, gender, educational level and cannabis use frequency. Enhancement, social, conformity and expansion motives showed no significant main effects. In all six models, PBSM-use significantly negatively predicted severity of dependence (-0.13 < βs < -0.11; CI -0.22 to -0.02), indicating that the more PBSM were used, the less symptoms of dependence were present, independent of covariates. Concerning moderation, only the interaction between routine motives and PBSM-use significantly negatively predicted severity of dependence (β = -0.1; CI -0.18 to -0.02). The effect of routine motives on the severity of dependence was 0.1 *SD* below average for individuals who were 1 *SD* above average in PBSM-use, independent of other motives, gender education and cannabis use frequency (see Fig 1). As depicted in Fig 2, routine motives were significantly related to the severity of dependence (0.66 < βs < 0.19) in individuals with relatively low to high use of PBSM (< 1.62 *SD* above the mean; 96.7% of all cases) and not significantly related in individuals with very high use of PBSM (> 1.62 *SD* above the mean; 3.3% of all cases).

## 4. Discussion

This study makes several important contributions to the understanding of the connection between cannabis use motives, protective behavior and dependence. For the first time the Protective Behavioral Strategies for Marijuana (PBSM) scale as well as the Marijuana Motives Measure (MMM) were used in a German speaking population. As done so before [31], we conceptualized PBSM as a possible mechanism to buffer the effects of distal antecedents of cannabis use and dependence. Therefore, beside the unique effects of PBSM and MMM, we explored their interaction in predicting severity of dependence.

Acceptable results of the confirmatory factor analysis of the PBSM scale as well as the good internal consistency correspond to the one-factor-structure found in a large sample of college students using advanced IRT techniques [25]. Also consistent with previous research, the PBSM score is significantly negatively correlated with cannabis use frequency and the severity of dependence, indicating that current users who employ PBSM more often show less cannabis use and lower severity of dependence. Most importantly, our sample differed to previous PBSM-research in that not only college students were assessed but a sample from the Swiss-germen speaking general population, diverse in age and educational level. We therefore propose that the use of PBSM functions as a protective factor not only in young adults but also across all age groups and educational levels. However, the model fit of the PBSM was not fully satisfying. This may be due to the translation process, a lack of cultural adaptation or the higher variability in use and success of PBSM by different age- or sociodemographic groups. It can also point to what was already observed during initial validation of the scale [24, 25]: After exploratory factor analysis, the initial information of the scree plot and eigenvalues included the possible solution of a multifactorial model. Understandably, it was dismissed after sound statistical procedures and conceptual thinking. However, it is possible that in our more diverse sample, differences in item functioning are more prominent than their shared aspects compared to homogenous groups of college students. It can be fruitful to look at the group-level differences of PBSM to deepen our understanding of how individuals from different groups control their cannabis use.

We used the extended version of the MMM, where routine motives had been added as a sixth factor containing the two items “out of boredom” and “out of habit” [36]. The CFA showed acceptable to good model fit and internal consistency. This result needs to be highlighted since the MMM has never been used before in a German speaking population and rarely in heterogeneous samples regarding age, educational level and cannabis use frequency. It opens the way to further investigations of cannabis use motives in German-speaking users. Routine motives appear to represent an important part of the MMM, given the model fit and moderate to strong positive correlations with cannabis use frequency and dependence. In line with Benschop et al. [36], this result emphasizes the importance of including routine motives in the investigation of cannabis use motives. However, since the factor contains only two items, it would be beneficial to explore if more items can be added to build a more reliable factor. Future studies should address this issue and refine culturally adapted versions of the MMM.

In all six moderation models, coping motives as well as routine motives significantly predicted severity of dependence, independent of gender, education, other motives and cannabis use frequency. For coping, this is in line with most existing research on the MMM [40, 50] and highlights their importance in dependent cannabis use patterns. The independent effect of routine motives suggests that this factor may play an equally important role in the progression from non-problematic to dependent cannabis use. It supports the argument made by Benschop et al. [36], that routine motives should be considered as a sixth factor in the MMM, given its robust correlation with cannabis use and its effect on dependence across age, education and use frequency. No other motives were predictive of severity of dependence. Still, their indirect influence on the severity of dependence should not be underestimated. In their recent meta-analysis, Bresin and Mekawi [41] found partial correlations of coping, enhancement and expansion motives with cannabis use frequency, which in turn is known to increase the risk for dependence; that is also reflected in our results.

Only routine motives interacted significantly with the use of PBSM in predicting severity of dependence. That suggests, the more often protective strategies are being used, the less strong routine motives are predictive of severity of dependence. This is in line with our assumption that the use of PBSM can be seen as a protective factor, moderating the effects of routine motives on cannabis dependence. However, if we look beyond *p*-values it becomes obvious that only at very high values of PBSM, or in 3.3% of the cases in our sample, does the effect of routine motives on the severity of dependence turn non-significant (see Fig 1), suggesting that PBSM-use exerts its protective function only if they are used extensively. This limits possible interpretation and further studies should identify specific subgroups for which the moderating effect of PBSM might be stronger.

In contradiction to our assumptions, there was no interaction between PBSM and coping motives in predicting severity of dependence. One possible explanation is that the connection between coping-driven cannabis use and experiencing psychological signs of dependence is too robust to be influenced by intuitively self-employed behavioral strategies. Similar to our results, Bravo et al. [31] found a negative interaction of coping motives and PBSM in predicting cannabis use frequency but not use-related negative consequences. Therefore, using PBSM may protect against frequent cannabis use in coping driven users but do not buffer their experience of dependence or other negative consequences. This perspective puts coping motives even more prominent as a risk factor for the progression from recreational cannabis use to dependence.

### 4.1. Limitations

Several limitations need to be mentioned. First, participants were recruited by letters sent to randomly chosen households in the city of Bern. We need to assume self-selection bias in form of an over-representation of participants who were highly interested in the topic, open to give information and enjoying reflecting on their cannabis use. Users who feel bad about their cannabis use might not want to think and give information about this issue. Therefore, the current results cannot be seen as representative of the general population, although the distribution of age, gender, educational level seemed to be satisfying. Second, our assessment of cannabis use frequency was problematic since it produces only nominal measures. It would have been beneficial to measure frequency of use in terms of “how many days per month” to gain interval scaled data and better comparability with other studies. However, as shown by the literature, the comparison between occasional, moderate and heavy users is meaningful and gives important insight into differences between those user groups.

### 4.2. Clinical implications

In this study, the main results on PBSM gathered in the US are replicated. The more often strategies are employed, the less often cannabis is used, and the less severe are symptoms of dependence. To reduce harm among current cannabis users, the promotion of PBSM as a self-regulation tool should be considered in prevention programs, in the educational context and as guidance for help-seeking current users. However, this might be the wrong approach for those with higher severity of dependence, like coping or routine users. The fact that we were not able to show a significant interaction effect between coping motives and PBSM implies that self-employed behavioral strategies do not work if cannabis is already used to deal with unwanted affective states. Although there was an interaction effect between PBSM and routine motives, the effect on the severity of dependence was limited to only a few cases who used PBSM extensively. We conclude that–in contrast to current users in general–PBSM are not applicable for dependent users, which is more likely the cases for coping and routine motives. Their protective effect seems to be limited to lower risk users. Users at risk must be addressed in another way, so that they understand the danger of coping and routine cannabis use. It is not clear how much PBSM protect current users against developing dependence over time and if training of PBSM has a positive effect on users who are already dependent. We encourage researchers to include PBSM and the MMM in prospective and intervention-studies to answer these open questions. This will help to understand the characteristics of non-dependent as well as dependent cannabis use patterns, so that users, policy makers and mental health professionals can understand, minimize and respond to harm associated with it.

## Supporting information

S1 Table Models with non-significant interactions. Bold = *p* < .05; β = z-standardized regression weights; PBSM = Protective Behavioral Strategies for Marijuana; Gender was coded male = 0 and female = 1. ^d^ = dummy-variable (the reference groups University degree and Occasional use were not entered in the model).
(DOCX)

S1 Appendix German translation of the Protective Behavioral Strategies Scale. Items are based on those published by Pedersen et al. [25].
(DOCX)

S2 Appendix German translation of the Marijuana Motives Measure. Items are based on Benschop et al. [36]. The numbering corresponds to the original item-list published by Simons et al. [33].
(DOCX)

S1 Dataset Imputed and pooled dataset with all variables included in the analysis. (XLSX)
