# Supplementary material for: Interactions of protective behavioral strategies and cannabis use motives: An online survey among past-month users
Source: PLoS One. 2021 Mar 1;16(3):e0247387. doi: 10.1371/journal.pone.0247387 (PMC7920385; doi:10.1371/journal.pone.0247387)
Supplement: S1 Table — Bold = p < .05; β = z-standardized regression weights; PBSM = Protective Behavioral Strategies for Marijuana; Gender was coded male = 0 and female = 1. d = dummy-variable (the reference groups University degree and Occasional use were not entered in the model). (DOCX) [file pone.0247387.s001.docx]

**S1 Table**

**Interaction models with non-significant interactions between Protective Behavioral Strategies for Marijuana (PBSM) and distinct cannabis use motives on severity of dependence.**

|  | β | SE | 95% CI | | |
| --- | --- | --- | --- | --- | --- |
| *Coping motives*  **Constant**  **Coping motives**  **PBSM**  Coping x PBSM  Enhancement motives  Social motives  Conformity motives  Expansion motives  **Routine motives**  Gender  High School degree^d^  Apprenticeship^d^  Higher College degree^d^  Moderate use^d^  **Heavy use^d^**  *Enhancement motives*  **Constant**  Enhancement motives  **PBSM**  Enhancement x PBSM  **Coping motives**  Social motives  Conformity motives  Expansion motives  **Routine motives**  Gender  High School degree^d^  Apprenticeship^d^  Higher College degree^d^  Moderate use^d^  **Heavy use^d^**  *Social motives*  **Constant**  Social motives  **PBSM**  Social x PBSM  **Coping motives**  Enhancement motives  Conformity motives  Expansion motives  **Routine motives**  Gender  High School degree^d^  Apprenticeship^d^  Higher College degree^d^  Moderate use^d^  **Heavy use^d^**  *Conformity motives*  **Constant**  Conformity motives  **PBSM**  Conformity x PBSM  **Coping motives**  Enhancement motives  Social motives  Expansion motives  **Routine motives**  Gender  High School degree^d^  Apprenticeship^d^  Higher College degree^d^  Moderate use^d^  **Heavy use^d^**  *Expansion motives*  **Constant**  Expansion motives  **PBSM**  Expansion x PBSM  **Coping motives**  Enhancement motives  Social motives  Conformity motives  **Routine motives**  Gender  High School degree^d^  Apprenticeship^d^  Higher College degree^d^  Moderate use^d^  **Heavy use^d^** | **-0.284**  **0.197**  **-0.118**  -0.037  -0.048  -0.078  -0.019  -0.010  **0.375**  -0.048  0.090  -0.009  -0.019  0.226  **0.462**  **-0.264**  -0.046  **-0.115**  0.007  **0.219**  -0.075  -0.021  -0.018  **0.376**  -0.052  0.088  -0.008  -0.015  0.215  **0.455**  **-0.266**  -0.073  **-0.114**  0.035  **0.219**  -0.045  -0.024  -0.017  **0.377**  -0.049  0.082  -0.003  -0.014  0.216  **0.458**  **-0.265**  -0.022  **-0.116**  0.004  **0.218**  -0.047  -0.076  -0.017  **0.376**  -0.052  0.089  -0.008  -0.015  0.214  **0.454**  **-0.264**  -0.017  **-0.116**  0.006  **0.218**  -0.047  -0.076  -0.021  **0.376**  -0.051  0.088  -0.008  -0.015  0.212  **0.454** | \| **0.116** \| \| --- \| \| **0.051** \| \| **0.047** \| \| 0.036 \| \| 0.046 \| \| 0.048 \| \| 0.042 \| \| 0.047 \| \| **0.050** \| \| 0.095 \| \| 0.138 \| \| 0.105 \| \| 0.112 \| \| 0.117 \| \| **0.132** \|   **0.115**  0.046  **0.048**  0.035  **0.047**  0.048  0.042  0.047  **0.050**  0.095  0.138  0.105  0.112  0.117  **0.132**  **0.114**  0.048  **0.047**  0.037  **0.047**  0.046  0.042  0.047  **0.050**  0.095  0.138  0.105  0.111  0.116  **0.131**  **0.115**  0.044  **0.048**  0.057  **0.047**  0.046  0.048  0.047  **0.050**  0.095  0.138  0.105  0.112  0.117  **0.132**  **0.115**  0.047  **0.047**  0.038  **0.047**  0.046  0.048  0.042  **0.050**  0.095  0.138  0.105  0.112  0.117  **0.131** | **-0.51**  **0.10**  **-0.21**  -0.11  -0.14  -0.17  -0.10  -0.10  **0.28**  -0.23  -0.18  -0.21  -0.24  0.00  **0.20**  **-0.49**  -0.14  **-0.21**  -0.06  **0.13**  -0.17  -0.10  -0.11  **0.28**  -0.24  -0.18  -0.21  -0.23  -0.02  **0.20**  **-0.49**  -0.17  **-0.21**  -0.04  **0.13**  -0.14  -0.11  -0.11  **0.28**  -0.24  -0.19  -0.21  -0.23  -0.01  **0.20**  **-0.49**  -0.11  **-0.21**  -0.11  **0.13**  -0.14  -0.17  -0.11  **0.28**  -0.24  -0.18  -0.21  -0.23  -0.02  **0.19**  **-0.49**  -0.11  **-0.21**  -0.07  **0.12**  -0.14  -0.17  -0.10  **0.28**  -0.24  -0.18  -0.21  -0.23  -0.02  **0.20** | **-0.06**  **0.30**  **-0.03**  0.03  0.04  0.02  0.06  0.08  **0.47**  0.14  0.36  0.20  0.20  0.46  **0.72**  **-0.04**  0.04  **-0.02**  0.08  **0.31**  0.02  0.06  0.07  **0.47**  0.13  0.36  0.20  0.20  0.44  **0.71**  **-0.04**  0.02  **-0.02**  0.11  **0.31**  0.05  0.06  0.08  **0.47**  0.14  0.35  0.20  0.20  0.44  **0.72**  **-0.04**  0.06  **-0.02**  0.12  **0.31**  0.04  0.02  0.07  **0.47**  0.14  0.36  0.20  0.20  0.44  **0.71**  **-0.04**  0.08  **-0.02**  0.08  **0.31**  0.04  0.02  0.06  **0.47**  0.14  0.36  0.20  0.20  0.44  **0.71** |  |

*Note.* Bold = p < .05; β = z-standardized regression weights; PBSM = Protective Behavioral Strategies for Marijuana; Gender was coded male = 0 and female = 1.

^d^ = dummy-variable (the reference groups *University degree* and *Occasional use* were not entered in the model).
