# Supplementary material for: Interactions of protective behavioral strategies and cannabis use motives: An online survey among past-month users
Source: PLoS One. 2021 Mar 1;16(3):e0247387. doi: 10.1371/journal.pone.0247387 (PMC7920385; doi:10.1371/journal.pone.0247387)
Supplement: S1 Appendix — Items are based on those published by Pedersen et al. [25]. (DOCX) [file pone.0247387.s002.docx]

**S1 Appendix**

**German translation of the Protective Behavioral Strategies Scale (PBSM-36)**

| *Bitte geben Sie an, wie oft Sie die unten genannten Strategien anwenden, wenn sie Cannabis/Marihuana einnehmen.*  1 = nie, 2 = selten, 3 = gelegentlich, 4 = manchmal, 5 = meistens, 6 = immer |
| --- |
| 1. Cannabis nur unter vertrauten Bekannten einnehmen. 2. Vermeiden, Cannabis einzunehmen, wenn Sie Zeit mit der Familie verbringen. 3. Vermeiden, vor der Schule/Arbeit zu konsumieren. 4. Vermeiden, Cannabis zu nutzen, um mit Emotionen wie Traurigkeit oder Niedergeschlagenheit umzugehen. 5. Kein Cannabis im Auto mitnehmen. Weder als Fahrer noch als Beifahrer. 6. Vermeiden, Cannabis an Orte oder Veranstaltungen mitzubringen, bei denen Sie durchsucht werden könnten. 7. Cannabis nur am Wochenende einnehmen. 8. Nach der Einnahme nicht Auto fahren. 9. Cannabis nur von einer vertrauten Quelle beziehen. 10. Vermeiden, Cannabis gewohnheitsmässig zu verwenden (jeden Tag oder mehrmals in der Woche). 11. Vermeiden, Cannabis vor dem Mittag einzunehmen. 12. Die Kosten im Auge behalten, um zu wissen, wie viel Sie für Cannabis ausgeben. 13. Vermeiden, Cannabis vor einer grossen Prüfung, Vorstellungsgespräch, Auftritt oder einer anderen wichtigen Aufgabe zu verwenden, bei der Sie klar sein müssen und bewertet werden. 14. Eine kleine Menge nehmen und dann abwarten, wie Sie sich fühlen, bevor Sie mehr nehmen. 15. Kein Cannabis kaufen. 16. Cannabis nicht mit anderen Drogen mischen. 17. Cannabis nur nachts verwenden (nicht während des Tages). 18. Aufhören Cannabis zu verwenden, wenn Sie ängstlich oder paranoid werden. 19. Vermeiden, Cannabis an öffentlichen Orten einzunehmen. 20. Regelmässige Pausen einlege, wenn Sie das Gefühl haben, zu oft zu konsumieren. 21. Weniger Cannabis auf einmal kaufen. 22. Situationen vermeiden, in denen Sie glauben zum Konsum gedrängt zu werden. 23. Nur dann einnehmen, wenn Sie wissen, dass für den Rest des Tages keine wichtigen Dinge zu erledigen sind. 24. Eine festgelegte Anzahl Züge (Stücke, Tropfen, etc.) nicht überschreiten (z.B. einen geteilten Joint weitergeben, wenn Sie diese Anzahl erreicht haben). 25. Vermeiden, Cannabis aus Langeweile einzunehmen. 26. Vermeiden, Cannabis so einzunehmen, dass Sie stärker berauscht sind als Sie möchten (z.B. Bong, Esswaren, Verdampfer, etc.). 27. Joints, Bongs etc. weitergeben, wenn Sie sich schon high fühlen. 28. höchstens ein Mal pro Tag/Nacht einnehmen. 29. Vermeiden, Cannabis in grösseren Zusammenkünften oder Menschenansammlungen einzunehmen. 30. Die Menge einer einmaligen Einnahme begrenzen. 31. Noch vor dem Ausgang (Party, Bar, etc.) entscheiden, ob Sie Cannabis einnehmen möchten oder nicht. 32. Vermeiden, Cannabis einzunehmen, wenn Sie sich ängstlich fühlen (z.B. um sich zu beruhigen oder Sorgen zu vertreiben). 33. Vermeiden, Cannabis in konzentrierter Form einzunehmen (z.B. Haschisch, Öl, Kif, Butter, etc.) um nicht "zu high" zu werden. 34. Längere Abstände der Einnahme oder 1-2-wöchtige Pausen machen, um die Toleranz zu senken. 35. Gerade genug verwenden, um einen leichten Rausch zu bekommen, ohne "zu high" zu werden. 36. Vermeiden, Cannabis vor physischer Aktivität (z.B. Workout, Wandern etc.) einzunehmen. |

*Note.* Items are based on those published by Pedersen et al. (2017).
