# Supplementary material for: Interactions of protective behavioral strategies and cannabis use motives: An online survey among past-month users
Source: PLoS One. 2021 Mar 1;16(3):e0247387. doi: 10.1371/journal.pone.0247387 (PMC7920385; doi:10.1371/journal.pone.0247387)
Supplement: S2 Appendix — Items are based on Benschop et al. [36]. The numbering corresponds to the original item-list published by Simons et al. [33]. (DOCX) [file pone.0247387.s003.docx]

**S2 Appendix**

**German translation of the Marijuana Motives Measure.**

| *Bitte geben Sie jeweils Sie an, wie oft sie aus einem der folgenden Gründe Cannabis konsumieren.*  1 = fast nie/nie, 2 = manchmal, 3 = die Hälfte der Zeit, 4 = meistens, 5 = fast immer/immer |
| --- |
| *Coping*  1. Um meine Sorgen zu vergessen.  4. Weil es mir hilft, wenn ich mich nervös oder niedergeschlagen fühle.  6. Um mich aufzuheitern, wenn ich schlechte Stimmung habe.  17. Um meine Probleme zu vergessen.  *Enhancement*  7. Weil ich das Gefühl mag.  10. Um high zu werden.  13. Weil es mir ein angenehmes Gefühl gibt.  18. Weil es Spass macht.  Social  3. Weil es mir hilft, Partys zu geniessen.  5. Um gesellig oder kontaktfreudig zu sein.  11. Weil soziale Anlässe/geselliges Beisammensein dann mehr Spass machen.  14. Weil dadurch Partys und Events besser werden.  15. Weil ich mich dann selbstbewusster und selbstsicherer fühle.  16. Um einen besonderen Anlass mit Freunden zu feiern.  *Conformity*  12. Um in die Gruppe zu passen, die ich mag.  19. Um gemocht zu werden.  20. Um mich nicht ausgeschlossen zu fühlen.  *Expansion*  21. Um mich selbst besser zu verstehen.  22. Weil es mir hilft, kreativer und origineller zu sein.  23. Um Dinge anders zu verstehen.  24. Um meine Wahrnehmung/mein Bewusstsein zu erweitern.  25. Um offener für Erfahrungen zu sein.  *Routine*  26. Aus Langeweile.  27. Aus Gewohnheit. |

*Note.* Items are based on Benschop et al. (2015). The numbering corresponds to the original item-list published by Simons et al. (1998).
